# A commentary on ‘causal effects of gut microbiota on renal tumor: a Mendelian randomization study’ (*Int J Surg* 2024 Jan 15; Online ahead of print.)

**DOI:** 10.1097/JS9.0000000000001249

**Published:** 2024-03-04

**Authors:** Junhao Chen, Jieming Zuo, Hongjin Shi, Haifeng Wang, Shi Fu

**Affiliations:** Department of Urology, The Second Affiliated Hospital of Kunming Medical University, Yunnan, People’s Republic of China

Highlights

- Conclusions drawn from a single cancer outcome database are not reliable.
- It is possible to further incorporate renal cancer outcomes from different databases and conduct meta-analysis of single nucleotide polymorphisms data.
- Further exploration should be conducted using the multivariable Mendelian randomization (MVMR) method to investigate exposures that may independently affect outcomes, separate from other gut microbiota.

*Dear Editor*,

We carefully read the article by Zhang *et al*.^1^ published in the International Journal of Surgery. They employed Mendelian randomization to assess the causal relationship between gut microbiota and renal tumors. The primary findings indicate that certain microbial taxa have a significant protective effect against benign and malignant renal tumors, while a minority may increase the incidence rate of these tumors. Additionally, there is a causal relationship between gut function and malignant renal tumors. This discovery contributes to a better understanding of the pathogenic mechanisms of gut microbiota in renal cancer and how to prevent it. We first express admiration for the authors’ outstanding work and innovative ideas. However, we have some thoughts to share on this topic.

Although the Mendelian randomization (MR) method can utilize genetic variations as instrumental variables to estimate the causal effects of exposure factors on outcome variables, it serves as an effective alternative strategy in inferring causality between exposure and outcome in the absence of randomized controlled trials (RCTs), using single nucleotide polymorphisms (SNPs) as instrumental variables (IVs). However, there are limitations in relying solely on conclusions drawn from a single cancer outcome database. Particularly in cancer research, due to the high biological specificity and heterogeneity of cancer, samples contained in a single database may not adequately represent the genetic backgrounds and environmental exposures of all relevant populations. Such limitations may lead to bias or inaccuracies in conclusions, thus affecting the correct inference of causality. The authors utilized renal cancer data from the Finn database. Therefore, to address the above issues, a feasible improvement strategy would be to incorporate renal cancer outcomes from different databases (e.g. ukb-b-1316 on the IEU website) as validation cohorts, while also conducting SNP meta-analysis. This approach can integrate data from various studies, increase sample size, enhance statistical power, and consequently obtain more robust and widely applicable conclusions. By meta-analyzing and combining information from different databases, this method can minimize sample selection biases and information biases that may exist in a single database, allowing for a more accurate estimation of the impact of exposure factors on specific cancer outcomes such as renal cancer.

Additionally, the authors identified certain microbial taxa from a large pool of gut microbiota that showed protective effects against both benign and malignant renal cancer. Although preliminary research has revealed correlations, we believe that these findings alone cannot comprehensively explain how gut microbiota independently influence specific outcomes. We argue that the complexity of the gut microbiome cannot be simply analyzed using a two-sample Mendelian randomization method, but rather more refined analytical methods should be employed to explore their independent effects. The MVMR method allows us to assess the independent effects of individual microbial taxa on health outcomes while controlling for other related microbial components. This method is particularly useful for specific microbial components that have direct effects on health outcomes in the presence of multiple exposure factors and interactions between exposures. It can facilitate a better understanding of microbiota-based therapies, prevention strategies, and further development of drug targets based on this point.

In summary, we extend our gratitude and admiration to Zhang *et al*. for their efforts in studying the causal relationship between gut microbiota and renal cancer. This article provides us with a preliminary understanding of the association and effects of gut microbiota on renal cancer. It lays a solid foundation for future in-depth research. However, while valuable and meaningful, we have expressed some concerns and areas for improvement to aid in conducting more effective related studies and advancing targeted therapies in the future.

## Ethical approval

Not applicable.

## Consent

Not applicable.

## Sources of funding

Not applicable.

## Author contribution

J.C. and S.F.: design, literature search, and writing; J.Z. and H.W.: literature search and data collections; H.S.: providing language polish.

## Conflicts of interest disclosure

The authors declare that they have no conflicts of interest. All data generated and analyzed during this study are included in this published article. The data presented in the article may be requested by consulting the correspondence author.

## Research registration unique identifying number (UIN)

Not applicable.

## Guarantor

Shi Fu.

## Data availability statement

The journal requires authors to include in any articles that report results derived from research data to include a data availability statement. Please confirm if any datasets generated during and/or analyzed during the current study are publicly available, available upon reasonable request, or if data sharing is not applicable to this article. None.

## Provenance and peer review

None.
